# 3D: diversity, dynamics, differential testing – a proposed pipeline for analysis of next-generation sequencing T cell repertoire data

**DOI:** 10.1186/s12859-017-1544-9

**Published:** 2017-02-27

**Authors:** Li Zhang, Jason Cham, Alan Paciorek, James Trager, Nadeem Sheikh, Lawrence Fong

**Affiliations:** 1 Division of Hematology and Oncology, Department of Medicine, UCSF Helen Diller Family Comprehensive Cancer Center, 550 16th Street, 6th Floor, UCSF Box 0981, San Francisco, CA 94158 USA; 2 Division of Hematology and Oncology, Department of Medicine, University of California, Room HSE301, UCSF Box 1270, 513 Parnassus Ave, San Francisco, CA 94143-1270 USA; 3 Department of Epidemiology and Biostatistics, University of California, San Francisco, 550 16th Street, 6th Floor, UCSF Box 0981, San Francisco, CA 94158 USA; 4 Research and Development, Nkarta, Inc, 329 Oyster Point Blvd, South San Francisco, CA 94080 USA; 5 Department of Research - Translational Biology, Dendreon Pharmaceuticals Inc, 1208 Eastlake Ave E, Seattle, WA 98102 USA

**Keywords:** Binary similarity measure, Caner immunotherapy, Clonality, Diversity index, Dynamics index, Differential testing, Fold change, Next generation sequencing, T cell receptor, T cell repertoire

## Abstract

**Background:**

Cancer immunotherapy has demonstrated significant clinical activity in different cancers. T cells represent a crucial component of the adaptive immune system and are thought to mediate anti-tumoral immunity. Antigen-specific recognition by T cells is via the T cell receptor (TCR) which is unique for each T cell. Next generation sequencing (NGS) of the TCRs can be used as a platform to profile the T cell repertoire. Though there are a number of software tools available for processing repertoire data by mapping antigen receptor segments to sequencing reads and assembling the clonotypes, most of them are not designed to track and examine the dynamic nature of the TCR repertoire across multiple time points or between different biologic compartments (e.g., blood and tissue samples) in a clinical context.

**Results:**

We integrated different diversity measures to assess the T cell repertoire diversity and examined the robustness of the diversity indices. Among those tested, Clonality was identified for its robustness as a key metric for study design and the first choice to measure TCR repertoire diversity. To evaluate the dynamic nature of T cell clonotypes across time, we utilized several binary similarity measures (such as Baroni-Urbani and Buser overlap index), relative clonality and Morisita’s overlap index, as well as the intraclass correlation coefficient, and performed fold change analysis, which was further extended to investigate the transition of clonotypes among different biological compartments. Furthermore, the application of differential testing enabled the detection of clonotypes which were significantly changed across time. By applying the proposed “3D” analysis pipeline to the real example of prostate cancer subjects who received sipuleucel-T, an FDA-approved immunotherapy, we were able to detect changes in TCR sequence frequency and diversity thus demonstrating that sipuleucel-T treatment affected TCR repertoire in blood and in prostate tissue. We also found that the increase in common TCR sequences between tissue and blood after sipuleucel-T treatment supported the hypothesis that treatment-induced T cell migrated into the prostate tissue. In addition, a second example of prostate cancer subjects treated with Ipilimumab and granulocyte macrophage colony stimulating factor (GM-CSF) was presented in the supplementary documents to further illustrate assessing the treatment-associated change in a clinical context by the proposed workflow.

**Conclusions:**

Our paper provides guidance to study the diversity and dynamics of NGS-based TCR repertoire profiling in a clinical context to ensure consistency and reproducibility of post-analysis. This analysis pipeline will provide an initial workflow for TCR sequencing data with serial time points and for comparing T cells in multiple compartments for a clinical study.

**Electronic supplementary material:**

The online version of this article (doi:10.1186/s12859-017-1544-9) contains supplementary material, which is available to authorized users.

## Background

T cells are a key component of the adaptive immune system, targeting infected or altered cells, such as cancerous cells. Cell targeting is a consequence of recognition of processed peptides displayed on the cell surface. Processed peptides are derived from antigens, presented by the major histocompatibility complex on target cells which in turn are recognized by the T cell receptor (TCR) on the surface of T cells [1]. In the context of cancer, antigens range from aberrantly expressed self-antigens to mutated self-antigens (neo-antigens) [2, 3]. Because of the enormous breadth of epitopes recognized by TCRs, the T cell repertoire is extremely diverse and dynamic. Diversity of the TCR is generated through somatic recombination during T cell differentiation in the thymus. Recombination of the Variable (V), Diversity (D) and Joining (J) antigen receptor segments, as well as stochastic nucleotide addition and deletions, in the TCR generate a hypervariable complementary determining region 3 (CDR3) – the portion of the TCR that mediates the specificity of peptide recognition [4–6].

The human immune system contains >10^9^ different T cells and measuring responses to immunotherapy by bulk biological analysis methods (e.g. flow cytometry) cannot sample enough T cells to characterize immunotherapy driven changes at the individual T cell clone level. The emergence of technologies such as next-generation sequencing (NGS) has allowed researchers to sequence across the variable region, which can be used as an identifier for T cell clonotypes. This allows researchers to track, and quantify, individual clonotypes across time as well as among different biological compartments such as circulating peripheral blood and intra-tumoral tissue [7] at a finer level than traditional assays such as flow cytometry [8]. This novel technology has recently been utilized to shed insight into the effects of immunotherapies such as anti-CTLA4 and anti-PD1 on anti-tumoral immunity and survival [9, 10]. It has also been leveraged to understand the heterogeneity of tumor infiltrating T cells and holds potential to be a prognostic biomarker [11, 12].

**Fig. 1:**
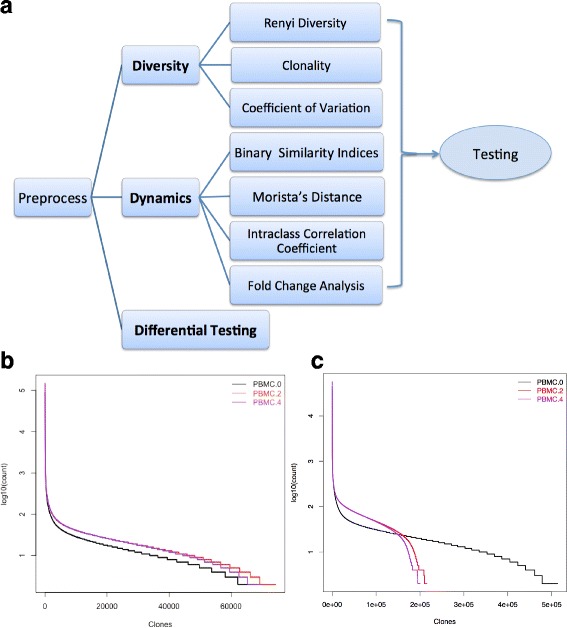
**a** The “3D” analysis pipeline of next-generation sequencing based TCR repertoire data. It consists of assessing the Diversity of the T-cell repertoire, evaluating the Dynamics of T-cell clonotypes across the time course or among different biological compartments, performing Differential testing to investigate differences in the abundance of each clonotype between pre- and post-treatment. **b** The count distribution of unique TCR clonotypes of a healthy subject (NeoACT study). Using one of the healthy subjects for illustration, the x-axis represents each unique clonotype in descending order of the count, and the y-axis is log_10_(count) of each clonotype from PBMC at week 0 (*black*), week 2 (*red*) and week4 (*purple*). **c** The count distribution of unique TCR clonotypes of a treated prostate cancer subject (NeoACT study)

Current approaches to understand the T cell repertoire diversity involve quantitating the number of unique clonotypes detected or utilizing ecological diversity indices such as the Shannon Index [13] and Clonality [14]. The Shannon Index and Clonality have been used to show that a more restricted T cell repertoire correlates with clinical response to pembrolizumab treatment in melanoma subjects [9, 15]. Recently, Cha et al. have utilized the Morisita’s Distance to assess the dynamics of the T cell repertoire and showed that repeated doses of anti-CTLA4 in melanoma and prostate cancer patients continued to remodel the T cell repertoire [10]. However, most literatures on TCR sequencing focus on the top ranked clones or the clones with larger abundance. Here, we proposed a “3D” analysis pipeline that was designed for assessing **Diversity** of the T-cell repertoire at a single time point, evaluating **Dynamics** of TCR sequencing across the time course or among different biological compartments, and performing **Differential testing** to detect the clonotypes whose abundance significantly changed among evaluated time points (Fig. 1a). We used the published data of an open-label, Phase II clinical trial of neoadjuvant sipuleucel-T [16, 17] and a Phase I/II clinical trial of ipilimumab with a fixed dose of GM-CSF to metastatic castration resistant prostate cancer patients [10] as the two test cases. Besides a detailed description of each measurement, we also examined the robustness of diversity/dynamics indices and compared their performance over the various thresholds used to filter the sequencing data. We then recommended major matrices for sample size calculation in a study where the diversity of T cell repertoire was one of the major endpoints. We further investigated the assessment of dynamic changes among different biological compartments by accounting for their presence or absence in each compartment assessed. Such an analysis pipeline will provide an initial workflow for TCR sequencing data with serial time points and/or in multiple compartments in a clinical context.

## Methods

Throughout this paper we define a sample as TCR sequencing data from a single biological sample of a subject at a particular time point. All the analyses were performed by R, the statistical computing software [18]. Statistical significance was declared at *p* < 0.05. Unless noted, there were no multiple testing adjustments performed. A typical TCR dataset for a single sample contains raw read count *f*_*i*_ and count frequency *p*_*i*_ for each clonotype, where *p*_*i*_ = *f*_*i*_/∑_*l*=1_^*n*^ *f*_*l*_. After preprocessing the raw sequencing data, for each sample, we first calculated the number of unique clones (*n*) and read depth *F* = ∑_*i*=1_^*n*^ *f*_*i*_, which is the measure of the total count of TCR sequences.

### Determination of TCR sequence diversity

We first characterized the diversity of clonotypes of each sample by using Renyi diversity of order *a*:

$$ {H}_a=\frac{1}{1- a} l o{g}_e{\sum}_{i=1}^n{p}_i^a, $$

where *p*_*i*_ is the frequency of clonotype *i* for the sample with *n* unique clonotypes, and the corresponding Hill number is *N*_*a*_ = exp(*H*_*a*_) [14]. As stated in [19], many common diversity indices are special cases of Hill numbers: *N*_0_ = n, *N*_1_ = exp(*H*), *N*_2_ = *D*_2_, and *N*_∞_ = 1/max(*p*_*i*_), where

$$ \begin{array}{l}\mathrm{Shannon}\ \mathrm{index}\ \mathrm{H}=-{\displaystyle \sum_{i = 1}^n}{p}_i\ { \log}_e\left({p}_i\right)\\ {} Gini\  Simpson\ {D}_1=1-{\sum}_{i=1}^n{p}_i^2\\ {} Inverse\  Simpson\ {D}_2=\frac{1}{{\displaystyle {\sum}_{i=1}^n}{p}_i^2}\end{array} $$

The Shannon index is a diversity index scaled from 0 to 1, minimally diverse to maximally diverse respectively. H/log_e_(*n*) is Pielou’s evenness (equability), and

$$ \mathrm{Clonality}=1-\mathrm{H}/{ \log}_{\mathrm{e}}(n), $$

which can be considered as a normalized Shannon index over the number of unique clones. Both Shannon index and clonality are the most popular indices currently used to assess T cell repertoire diversity. We can regard a sample more diverse if all of its Renyi diversities are higher than in another samples.

We also considered coefficient of variation (CV), known as relative standard deviation, to assess the TCR diversity. It is a standardized measure of dispersion of a probability distribution or frequency distribution and was first used to assess the TCR diversity in Dziubianau et al. [20]. Since the frequency distribution of the TCR sequence was skewed to small frequencies (Fig. 1b and c), we considered logarithm transformation with base 10 of clonotypes’ frequency, i.e., *log*_10_ *p*_*i*_, therefore, we used geometric coefficient of variation (GCV) defined by Kirkwood [21]:

$$ \mathrm{G}\mathrm{C}\mathrm{V}= \exp \left({S}_{ln}-1\right), $$

where *S*_*ln*_ = *S* × 10 × *log*_*e*_(10) and *S* is the standard deviation of *log*_10_ *p*_*i*_, *i* = 1, …, *n*.

### Evaluation of the dynamic nature in TCR sequence across time or between different biological compartments

To assess the dynamic nature in TCR repertoire, we measured the overlap among TCR sequences across time points or between different biological compartments for the same subject by binary similarity matrices. Choi and the coauthors [22] collected 76 binary similarity measures used over the last century and revealed their correlations through hierarchical clustering technique. As an example, we utilized the Baroni-Urbani and Buser (BUB) overlap index [23]. Unlike most of the overlap index measures, BUB includes the negative matches, i.e., the absent clones. For example, to calculate BUB of each two time points across three time points *j*_*1,*_ *j*_*2*_ and *j*_*3*_, we first consolidated all clones present in any of the three time points and let *n*_1_ = the number of clones present at time *j*_*1*_;*n*_*2*_ = the number of clones present at time *j*_*2*_; *n*_*12*_ = the number of clones present in both time points and *d*_*12*_ = the number of clones absent in both time points; then BUB overlap index of time points *j*_*1*_ and *j*_*2*_ equals:

$$ B U{B}_{j_{1{j}_2}}=\frac{n_{12}+\sqrt{n_{12}{d}_{12}}}{n_1+{n}_2-{n}_{12}+\sqrt{n_{12}{d}_{12}}}. $$

It is equivalent to the Jaccard coefficient = $\frac{n_{12}}{n_1+{n}_2-{n}_{12}}$, when there are only two time points. The advantage of BUB overlap index is that it includes the information of the number of the absent clones, thus allows the researchers to observe and account for changes across all available samples. This ensures that different paired BUBs (e.g. *BUB*_12_, *BUB*_13_ and *BUB*_23_) across the same set of available samples are comparable. There are several other binary similarity measures that have closer distance with the BUB overlap index based on hierarchical clustering, thus can be considered as the substitute of the BUB overlap index, such as $B U{B}_2=\frac{3{n}_{12}-\left({n}_1+{n}_2\right)+\sqrt{n_{12}{d}_{12}}}{n_1+{n}_2-{n}_{12}+\sqrt{n_{12}{d}_{12}}}$, Faith and Mountford [22].

The binary similarity measures are straightforward but only use very limited information of TCR repertoire, i.e., the presence or absence of clones across the samples. In addition, we utilized the relative clonality (RCL) which was calculated as the ratio of the clonality at two time points to measure the dynamics. Furthermore, we considered matrices which aggregate the changes in abundance of each clonotype across time points to evaluate the dynamic nature of TCR repertoire across time course. Morisita's overlap index [24] has been used in several recent publications as a statistical measure of dispersion of clones in TCR sequence [10]. It is based on the assumption that increasing the size of the samples will increase the diversity because it would include more different clonotypes.

$$ {C}_D=\frac{2{\displaystyle {\sum}_{i=1}^m}{f}_{i j}{f}_{i k}}{\left(\frac{{\displaystyle {\sum}_{i=1}^m}{f}_{i j}^2}{F_j^2}+\frac{{\displaystyle {\sum}_{i=1}^m}{f}_{i k}^2}{F_k^2}\right){F}_j{F}_k} $$

*f*_*ij*_ and *f*_*ik*_ are the abundance of clonotype *i* with the read depth *F*_*j*_ and *F*_*k*_ from time point *j* and *k*, respectively. *C*_*D*_ = 0 if the two samples do not overlap in terms of clonotypes, and *C*_*D*_ = 1 if the clonotypes occur in the same proportions in both samples.

The intraclass correlation coefficient (ICC) is another matrix we proposed to evaluate dynamic nature in clone abundance, which is commonly used to quantify the degree to which individuals with a fixed degree of relatedness resemble each other in terms of a quantitative trait. One of the applications of ICC is to assess the persistence of quantitative measurements at different time points for the same quantity. In the framework of a random effects models *z*_*ij*_ = *u* + *a*_*j*_ + *e*_*ij*_, where *z*_*ij*_ = *log*_10_ *p*_*i*_ of the observed clone *i* in sample *j* for a particular subject, *u* is an unobserved overall mean, *a*_*j*_ ~ *N*(0, *S*_*a*_^2^ ) is an unobserved random effect shared by all clones in sample *j*, and *e*_*ij*_ ~ *N*(0, *S*_*e*_^2^) is an unobserved random error. Both *a*_*j*_ and *e*_*ij*_ are assumed to be identically distributed, and uncorrelated with each other. Thus,

$$ I C C=\frac{S_a^2}{S_a^2+{S}_e^2}. $$

The function ‘icc’ in R package ‘irr’ [18] was used to calculate ICC. The advantage of ICC is that it can be used to evaluate the dynamic change in clone abundance for more than 2 time points. However, due the nature of the TCR sequences that a big proportion of clones only present at one time point, i.e., their counts equal 0 in another time points, which greatly drives the value of ICC. Therefore, ICC is more appropriate to evaluate the dynamic change of the common clones present at all the time points that we are interested in.

Besides aggregating the dynamic changes of clones of the T cell repertoire, we further investigated the distribution of the fold change (FC), for clonotype *i*, $F C={ \log}_2\frac{p_{ik}}{p_{ij}}$, where *k* and *j* are two different TCR samples from the same subject. Furthermore, based on FC, we clustered the clonotypes into three groups: decrease if FC ≤ -c, unchanged if –c < FC < c and increase if FC ≥ -c, where *c* is an arbitrary constant, for example *c* = 2 stands for a 4-fold change. When comparing the clonotypes frequencies between different biological compartments (e.g., blood sample and tissue sample), we recommended adjustment to account for the distinctions due to the biological characteristics. For example, we multiply c by ∑_*i*=1_^*m*^ *log*_2_ *p*_*ik*_/∑_*i*=1_^*m*^ *log*_2_ *p*_*ij*_.

### Exploration of the treatment effect or the clinical benefits

As stated above, to explore the treatment effect or the clinical benefits, the diversity/dynamics index can be served as an endpoint. To test for a treatment effect, we can compare the diversity index of all subjects among time points by repeated measures analysis of variance (ANOVA) (or its nonparametric comparative). To explore the difference of over-time dynamics among the groups defined by clinical outcomes (e.g., clinical responders vs. non-responders or long-term survivors vs. short-term survivors), we can compare the dynamics index among the groups by ANOVA (or its nonparametric comparative). In addition, to allow for a varying number of follow-up measurements, the repeated measure ANOVA methods with a mixed model approach (treating time as a random effect and clinical outcome as a fixed effect) can be utilized, and the specific comparison of change in the diversity index between baseline and any specific post-baseline time point can be tested using linear contrast.

### Differential testing

The methods described above treated all clonotypes from the same sample as a single unit, and therefore failed to distinguish which unique clonotypes may be the most significant driver for observed effects. We therefore considered a modified differential expression analysis (DEseq) [25] to explore treatment effects on the abundance of clonotypes for each clonotype as we did in our recent work [10]. The DESeq R package [25] was developed explicitly for identification of differentially expressed genes in RNA-Seq experiments and it is technically possible to work with experiments with small number of replicates or without any biological replicated. TCR repertoire data differs from typical gene expression data, in that it is heavily skewed towards rare clonotypes, with large numbers of clonotypes appearing only a few times, and many clonotypes appearing only once [10]. Modifications were made to accommodate the specific case of repertoire analysis: 1) normalization was performed using only clonotypes that had > =5 counts in at least one sample; 2) a dispersion model calculated as the median of dispersion curves from all samples (more detailed illustration in the result section). This modification served to account for normal variation in the repertoire over time, and to compensate for the lack of replicates in the experimental design. The detection of the significant clones by DESeq analysis was based on controlling for false discovery rate (FDR) [26] <0.05.

### Illustration datasets

TCR profiling data from five subjects enrolled in the NeoACT study (NCT00715104) [16, 17] were used for major illustration. NeoACT study was a phase II neoadjuvant study examining whether sipuleucel-T induced T cell infiltration into the prostate. Subjects received sipuleucel-T (prepared by culturing freshly obtained leukapheresis peripheral blood mononuclear cells (PBMC) with a fusion protein of prostatic acid phosphatase and GM-CSF) at the standard 2-week intervals for three planned doses. Radical prostatectomy was performed 2–3 weeks after the final sipuleucel-T infusion. PBMCs were evaluated in the five treated subjects at week 0 (before sipuleucel-T treatment) and during treatment at weeks 2 and 4. RP tissues from the same subjects were also evaluated. In addition to the NeoACT subjects, TCR data from three healthy donors and five untreated prostate cancer subjects were also used for comparative purposes. Serial (week 0, 2 and 4) PBMCs from healthy subjects receiving no treatment as well as PBMC and RP tissue from untreated prostate cancer subjects were used as comparators.

The second dataset includes PBMCs from 21 metastatic castration resistant prostate cancer patients treated with anti-CTLA-4 (ipilimumab) and GM-CSF in a single-center phase I/II clinical trial (NCT00064129) [10]. Patients were treated with up to four doses of ipilimumab ranging from 1.5 to 10 mg/kg and GM-CSF at 250 mg/m2 per day. Anti–CTLA-4 antibody was administered every 4 weeks with GM-CSF given daily on the first 2 weeks of these cycles. Only baseline (week 0) and week 2 data were included in the current paper for illustration purpose (results/figures were presented in the Additional file 1: Figure S6).

### TCRβ amplification and sequencing

The TCRβ CD3 (CDR3β) region for both PBMC and tissue samples was amplified and sequenced using the ImmunoSEQ assay (Adaptive Biotechnologies). The amplification and sequencing of TCRβ repertoire as well as clonotype identification and enumeration have been previously described in detail [27].

## Results

### Visualization of TCR sequence abundance before and after sipuleucel-T treatment

Instead of using scatter plots, which are commonly used to visualize the distribution of frequencies of two TCR samples from the same subject, we plotted the log_10_(count) of each unique clonotype in descending order of count (Fig. 1b, c), and inclusive of multiple samples in one graph. The distributions of clonotype frequencies of serial blood samples obtained every 2 weeks were very similar in a healthy subject (Fig. 1b). Whereas the prostate cancer subject receiving sipuleucel-T treatment had different distribution profiles among the three time points (Fig. 1c). We also observed that the baseline curve intersected with the curves at week 2 and week 4 at count of 23 (log_10_(count) = 1.36) and 24 (log_10_(count) = 1.36), respectively. The similar results were found for other treated patients (figures were not shown) with the intersection points ranging from count of 10–30, which implied that the difference in the number of unique clones was caused by the clones with the counts smaller than those intersection points. The clones with counts smaller than the intersection point might have influence on the diversity and dynamics indices; therefore, those intersection points might be helpful for finding the best cutoff to filter the data. Our R package provides the function to obtain such an intersection point.

### TCR sequence diversity changed following the first treatment with sipuleucel-T

The first phase of the proposed “3D” analysis pipeline was quantifying diversity (Additional file 2: Figure S1A-C). As shown in Additional file 2: Figure S1B, the clonality for the healthy subjects were consistent for two subjects across time with the third subject was later verified having a cold at week 0. The treated subjects had a wide range of baseline clonality, however, the clonality of the majority of treated subjects had a decrease from week 0 to week 2 (*p* = 0.063) but became stable from week 2 to week 4 (*p* = 0.875) indicating that TCR diversity changed after the first treatment but didn’t significantly change from week 2 to week 4.

### Evaluation of the dynamics of TCR sequence across the sipuleucel-T treatment time course showed that the commonality of TCR sequence between week 2 and 4 increased

As presented in Additional file 3: Figure S2A, the BUB overlap indices of PBMC over week 0, 2 and 4 were consistently about 0.2 for healthy donors, but for the treated prostate cancer subjects there was a significantly greater increase in the overlap between week 2 and 4 than the overlap of week 2 (week 4) with baseline (*p* = 0.004). Additional file 3: Figure S2B show that the healthy subjects had a consistent ICC of 0.8, however, the treated subjects had much higher ICC at week 2 with week 4 than that of baseline with either week 2 or week 4 (*p* = 0.011 and *p* = 0.008, respectively). This demonstrated that for the treated subjects when compared to baseline PBMC, PBMC samples at week 2 and week 4 had greater concordance, confirming an immediate sipuleucel-T treatment effect.

**Fig. 2:**
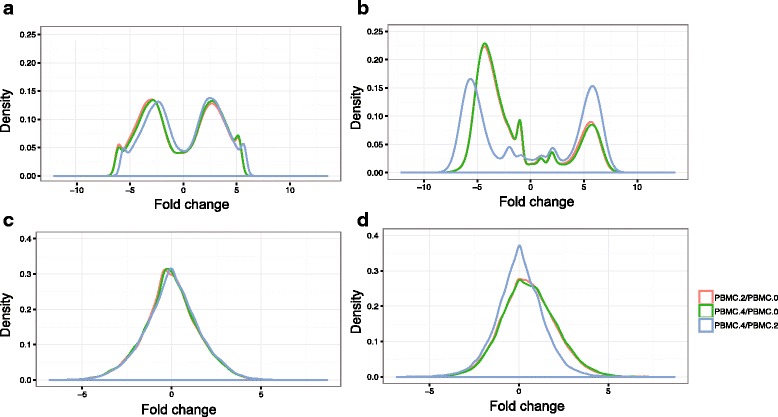
The distribution of the pairwise fold change (FC) between PBMC samples (NeoACT study) for one healthy subject (**a**, **c**) and one treated prostate cancer subject (**b**, **d**). For clonotype *i*, FC is calculated by $F C={ \log}_2\frac{p_{ik}}{p_{ij}}$, where *k* and *j* are the samples from two different time points for the same subject. Each curve represents a pair of samples: PBMC.2 vs. PBMC.0 (*red*), PBMC.4 vs. PBMC.0 (*green*) and PBMC.4 vs. PBMC.2 (*blue*). Top figures (**a**, **b**) include the clones present at either of the sample from a pair and bottom figures (**c**, **d**) include the clones present at both samples from a pair (i.e., the overlap clones)

The three FC distribution curves (PBMC week 2/week 0, week 4/week 0 and week 4/week 2) of the healthy subjects had a similar pattern (Fig. 2a, c), whereas for treated subjects there was a large shift in the week 4/week 2 FC curve compared to other two curves (Fig. 2b, d). We further calculated the proportions of decrease/unchanged/increase in terms of clone frequency by setting c = 2. There was a significant increase in the proportion of unchanged clones between week 2 and week 4, and a significant drop in the proportion of increased clones from week 2 to week 4 (Additional file 3: Figure S2C). This indicated that from baseline to week 2 and week 4, about 15–25% of the overlapped clone abundance was enriched and this enrichment remained from week 2 and week 4. FC analysis further implied that the immediate sipuleucel-T treatment effect might enrich the abundance of a certain group of clonotypes.

### Assessment of dynamic changes from PBMC to tissues revealed that RP tissues became resemblance with week 2 and week 4 PBMC after sipuleucel-T treatment

**Fig. 3:**
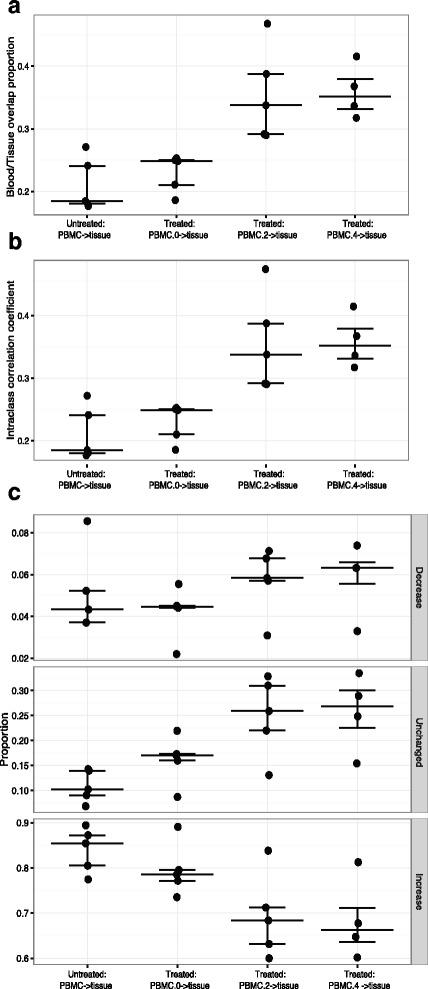
The dynamics from PBMC to tissue for prostate cancer subjects (NeoACT study). **a** The proportion of overlap between PBMC and RP tissue. The traditional formula was used to calculate the overlap proportion of T-cell clonotypes between RP tissue and PBMC at each time point (PBMC.0- > tissue, PBMC.2- > tissue, PBMC.4- > tissue) for the treated prostate cancer subjects and untreated subjects (PBMC- > tissue). **b** The intraclass correlation coefficient (ICC) between RP tissue and PBMC. The ICC was calculated based on the clones present at both RP tissue and PBMC from the untreated prostate cancer subjects (PBMC- > tissue), or between RP tissue and PBMC at each time point of the treated prostate cancer subjects (PBMC.0- > tissue, PBMC.2- > tissue, PBMC.4- > tissue). **c** The binned analysis of fold change in clonal frequency from PBMC to RP tissue. This fold change analysis only included the clones that present at both tissue and PBMC for the untreated subjects (PBMC- > tissue) or present at both tissue and PBMC at each week (PBMC.0- > tissue, PBMC.2- > tissue, PBMC.4- > tissue), respectively, for the treated prostate cancer subjects. From top to the bottom, each panel presents the fraction of the decrease, unchanged and increase clones which correspond to the adjusted FC of tissue vs. PBMC is less than 0.25, between 0.25 and 4 and greater than 4, respectively. The median and interquartiles are shown

Our previous finding showed that the TCR sequence diversity within RP tissue was significantly higher in subjects who received sipuleucel-T treatment compared to untreated prostate cancer subjects (*p* = 0.01). To explore the dynamic change of clonotypes from PBMC to RP tissue, we calculated the proportion of overlap (Jaccard coefficient) between tissue and PBMC at each time point separately for both treated and untreated subjects. Similar overlap proportions between tissue and PBMC were observed for the untreated subjects and for that of the treated subjects at baseline (*p* = 0.158), but a greater increase was seen between tissue and PBMC week 2 or week 4 for the treated subjects (*p* = 0.008 and 0.016, respectively) (Fig. 3a).

Comparing to the untreated subjects (Fig. 3b), ICCs of week 0 PBMC and tissue of the treated subjects were similar (*p* = 0.310), but ICC of week 2 or week 4 PBMC with tissue dramatically increased (*p* = 0.008 and 0.016, respectively). Moreover, comparing with the untreated subjects (Fig. 3c), there was a significant increase in the proportion of unchanged clones from week 2 or week 4 PBMC to the tissue for the treated subjects (*p* = 0.032), which implied that RP tissue resembled at week 2 and week 4 PBMC for those clones present constantly. There was a significant drop in the proportion of increased clones from week 2 (or 4) PBMC to the tissue (60–84%) when compared to week 0 PBMC vs. tissue (74–89%) (p = 0.032), indicating about 5–20% of the overlap clones in RP tissue were enriched immediately after the first treatment. These implied that sipuleucel-T treatment increased TCR sequence commonality between blood and resected prostate tissue in the treated subjects comparing to the untreated subjects.

### DESeq analysis demonstrated sipuleucel-T treatment induction of that were present in the prostate tissue

**Fig. 4:**
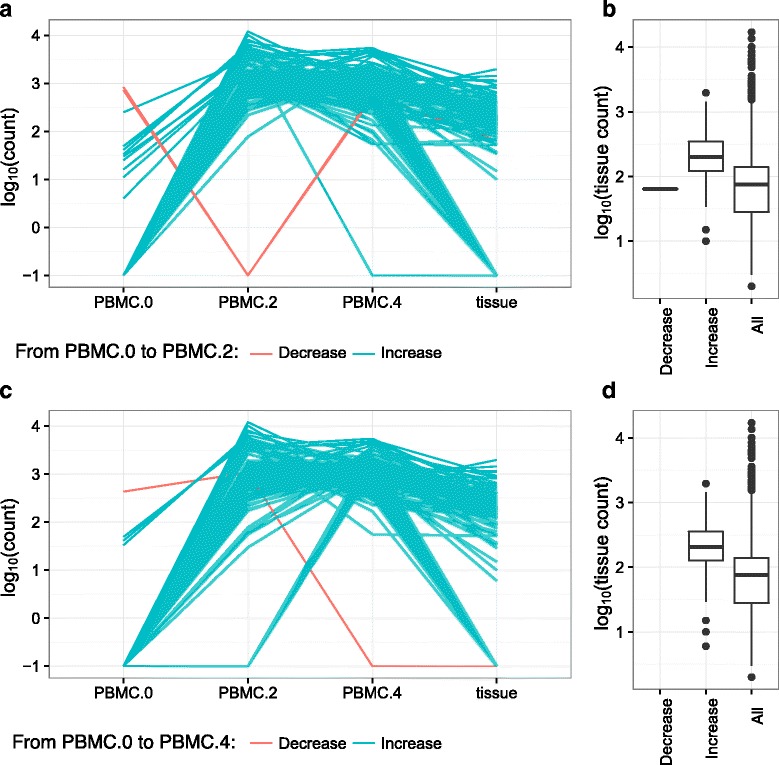
Significantly differentiated clones detected by DESeq analysis for one treated prostate cancer subject in NeoACT study (FDR < 0.05). **a** Tracking plot of the 127 clones that were significantly changed from week 0 to week 2. *Green* and *red* lines represent the increased and decreased clones from baseline PBMC to post-treatment. **b** Boxplots of log_10_ of tissue T-cell repertoire clonotype count for the 83 tissue-present clonotypes that were also significantly changed from week 0 to week 2. The left and the middle boxplots present log_10_(tissue count) of the clones significantly decreased (*n* = 1) or increased (*n* = 82) from baseline to post-treatment, respectively. The right plot presents all tissue-present clones. **c** Tracking plot of the 135 clones that were significantly changed from week 0 to week 4. *Green* and *red* lines represent the increased and decreased clones from baseline PBMC to post-treatment. **d** Boxplots of log_10_ of tissue T-cell repertoire clonotype count for the 89 tissue-present clonotypes that were also significantly changed from week 0 to week 4. The left and the middle boxplots present log_10_(tissue count) of the clones significantly decreased (*n* = 0) or increased (*n* = 89) from baseline to post-treatment, respectively. The right plot presents all tissue-present clones

For each treated subject, we first calculated the dispersion based on each pair of the PBMC samples and performed 1 to 1 comparison by modified DESeq (**1 vs. 1** in Additional file 4: Table S1). Next we calculated dispersion on all PBMC samples, and performed pairwise comparison (**All Samples** in Additional file 4: Table S1), and then compared PBMC at week 2 and 4 with PBMC at baseline. We found, for example, within the treated subject 24, 127 clones were significantly changed from week 0 to week 2 (FDR < 0.05), of which 83 (65.4%) of clones were present in the tissue (Fig. 4a). Comparing log_10_(tissue count) of the 82 significantly enriched clones from week 0 to week 2 which also presented in tissue with mean of log_10_(tissue count) of all 22350 tissue-present clones (Fig. 4b), we found that these 82 significantly enriched tissue-present clones had significantly higher tissue count than the overall mean (*p* < 0.001), supporting the hypothesis that sipuleucel-T induces extravasation of T-cells into the prostate tissue. We also detected 135 clones significantly changed from week 0 to week 4 (FDR < 0.05), of which 89 (65.9%) of clones were present in the tissue (Fig. 4c), and the tissue count of those 89 clones also had significantly higher tissue count than the overall mean (*p* < 0.001). Similar results were observed for the other sipuleucel-T treated subjects (Additional file 4: Table S2).

## Discussion

The proposed analysis pipeline is designed to investigate two major aspects of the T cell repertoire: diversity and dynamics, and further perform differential testing for each clone. Here, a diversity index reflects how much difference among the TCR repertoire within each sample, while the dynamics analysis is to evaluate clone abundance change across the samples for the same subject, moreover, differential testing aims to detect the single clonotypes that have significantly different abundance across samples for the same subject. A public available R software “TCR3D” (https://github.com/mlizhangx/TCR-3D) is developed to implement the proposed workflow.

Based on the preprocessed TCR repertoire data (which is out of scope of the current paper), starting with obtaining the number of unique clones and read depth for each sample, we suggest first assessing the repertoire diversity. Although Clonality is recommended, calculating more than two diversity measures is highly recommended to ensure consistent results and a sample can be considered more diverse if all of its Renyi diversities (Hill numbers) are higher than in another samples [14]. The number of unique clones and read depth should not be considered as the basis for an overall conclusion. If a study has multiple observations available for the same subject - usually obtained at different time points (e.g., before and after treatment), then dynamics analyses, such as evaluation of binary similarity measures, morisita’s distance, ICC, etc., and fold change analysis, are expected. In addition, when assessing commonality between different biological compartments consideration of the inherent variation due to the different biological mechanism is highly recommended, such as adjusting the clone frequency by the ratio of read depth, though we readily acknowledge that more advanced work (such as computer simulation study) might be warranted to further address this issue. Note each analysis component is performed for each single subject separately, to obtain meaningful scientific inference, we need to further compare the index between different time points or between different patient groups (Additional file 1: Figure S6A-C) with a valid statistical test. Furthermore, differential testing needs to be taken into consideration with necessary modification on normalization and dispersion estimation, especially when replicates are available. DESeq was applied solely for the illustration purpose. It has been developed to enable analysis of experiments with small number of replicates and it is technically possible to work with experiments without any biological replicated, which meets our situation that the differential testing of TCR data can only be done within each subject and there are very limited or no biological replicates within each subject. Seyednasrollah et al. [28] summarized and compared the software packages for detecting differential expression and stated that other existing methods to test differential expression require relative larges number of replicate samples. However, most of the softwares are applicable in R environment [18], thus are compatible with our developed R package.

Though there are a number of methods and software available for immunoglobulin (IG) and TCR profiling (Additional file 5: Table S3) [29], these computational methods were mainly used for processing repertoire data by mapping V, D, J antigen receptor segments to sequencing reads and assembling T- and B-cell clonotypes, and most of them are not designed to quantify the diversity and dynamics of the repertoire. For example, miXCR [30] is a universal framework that processes big immunome data from raw sequences to quantitated clonotypes. The more comprehensive software, LymAnalyzer [31], consists of four functional components: VDJ gene alignment, CDR3 extraction, polymorphism analysis and lineage mutation tree construction. sciReptor [32] is a flexible toolkit for the processing and analysis of antigen receptor repertoire sequencing data at single-cell level by a relational database. Some of the tools, such as repgenHMM [33], IMonitor [34], IMEX/IMmunEXplorer [35], Change-O [36], ImmunediveRsity [37], and VDJtools [38] etc., could also measure repertoire diversity, but they only rely on one or two diversity indices, such as Shannon or Gini diversity. ImmunoSEQ Analyzer [39] developed by Adaptive Biotechnologies, a pioneer in leveraging NGS to profile T- and B-cell receptors, provides web-based analysis for TCR data including estimation of diversity and dynamics indices, though with limited options; and unfortunately, it is only available to the customers who have sequencing performed by Adaptive Biotechnologies. Recently, Nazarov et al. [40] developed an R package “tcR” to analyze NGS-based T cell repertoire data, that integrated widely used methods for individual repertoires analyses and TCR repertoires comparison, customizable search for clonotypes shared among repertoires, spectratyping, and random TCR repertoire generation. However, both immunoSEQ Analyzer and the “tcR” package do not provide detailed discussion about the robustness of diversity/dynamic indices, lacks the ability to investigate the unique dynamic nature of this type of sequencing data, especially between different types of biological compartments and don’t offer the feature of differential testing of each individual clone.

**Fig. 5:**
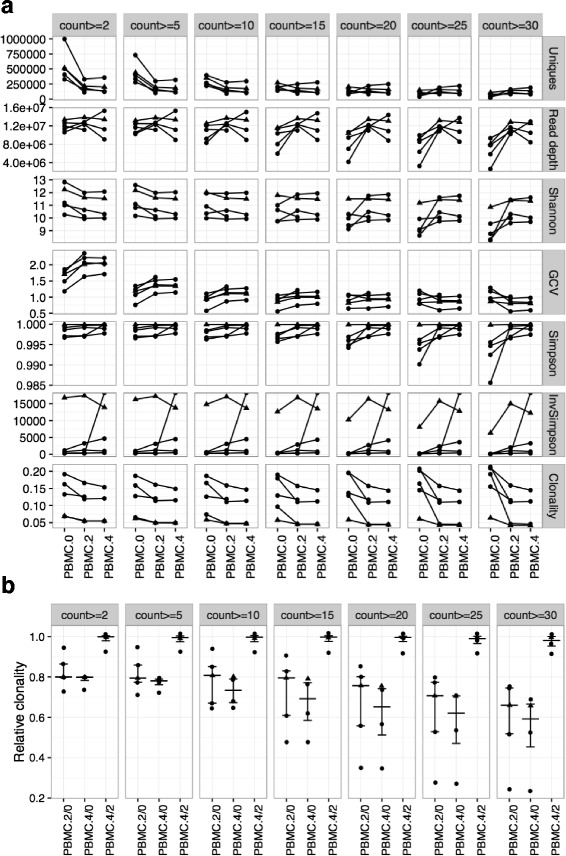
The influence of the count thresholds on diversity matrices of TCR repertoire in sipuleucel-T treated prostate cancer patients (NeoACT study). **a** TCR sequencing data of PBMC samples at week 0 (PBMC.0), week 2 (PBMC.2) and week 4 (PBMC.4) of the five treated prostate cancer subjects are used for illustration. From top to bottom, each row shows the number of unique clones (Uniques), read depth, the Shannon index, Gini Simpson, Inverse Simpson (InvSimpson), geometric coefficient of variation (GCV) and Clonality of TCR repertoire. From the left to the right, each column presents the different threshold of the clonotypes count (original data which is > =2, > = 5, > = 10, > = 15, > = 20, > = 25 and > =30). The Shannon index, Clonality, Gini Simpson, Inverse Simpson and GCV were obtained by recalculating the clone frequency after filtering the data with the different cutoffs. **b** Pairwise relative clonality were calculated as the clonality of PBMC at the later time point divided by that of the earlier time point, e.g., PBMC.2/0 = clonality of PBMC Week 2 divided by PBMC Week 0. From the left to the right, each column presents the different threshold of the clonotypes count (original data which is > =2, > = 5, > = 10, > = 15,> = 20, > = 25 and > =30). The subject with triangle shapes was the example used in Fig. 1c)

We examined the robustness of diversity/dynamics indices with the number of unique clones whose differences were mainly driven by low-count clones, and compared the performance of the diversity/dynamics indices over the various thresholds used for filtering the sequencing data (Additional file 6: Document). We found that Clonality and relative clonality were the matrices that possessed robustness to different count thresholds (Fig. 5), the binary similarity measures were greatly influenced by the lower count clones (Additional file 7: Figure S4), and Morisita’s distance had better performance when TCR repertoire only retains the high abundance clones (Additional file 8: Figure S5). Furthermore, we also performed differential testing on the clones with different thresholds (detailed results were not shown), which show that more than 86% of clones detected significant when applying a threshold of count ≥ 5 were still detectable when applying other thresholds (count ≥ 10 ~ 30). Currently, the TCR data from the vendors (Adaptive Biotechnologies or other sequencing companies) all have their own preprocessing steps which may be proprietary. However, we advocate not just working on the top ranked clones, such as the clones with the count in top 25%, or the clones with larger abundance (count ≥ 50), but rather considering possible but necessary filtering on the data to avoid the potential noises caused by low-count clones and performing robustness check

TCR diversity and dynamics might someday be used as predictive biomarkers in cancer immunotherapy. Therefore, we propose that if testing the treatment effect is the primary objective, sample size calculation should be based on a paired *t*-test or repeated measures ANOVA of the diversity index, where Clonality is recommended; if examining the influence of the clinical outcome (such as the clinical response to the treatment) is the major goal, sample size calculation should be based on a two-sample *t*-test or ANOVA of the dynamic index (BUB or relative clonality is recommended).

To extend the pipeline, in our next step, we would perform both manual and automated approaches in biological annotation such as summarizing the V, D, J gene families used to construct the TCR to further explore the biology of the T cell repertoire. Both supervised and unsupervised clustering clonotypes within a sample or across different time points is part of our future work too, though we recognize that due to the large number of clonotypes and low overlap caused by dynamic feature of the TCR sequencing data, finding a suitable distance measure and an efficient clustering method is a challenging task.

## Conclusions

By using the proposed “3D” analysis pipeline to the real example, we were able to evaluate the TCR sequence diversity of each sample and investigated the changes in abundance of each clonotype across time and between blood and tumor tissue. Through this approach, we discovered that sipuleucel-T treatment changed the TCR repertoire in the blood and in prostate tissue. We also found that the increases in common TCR sequences between RP tissue and blood after sipuleucel-T treatment supported the hypothesis of a treatment-induced T cell migration into the prostate tissue. The pipeline is a thorough analysis of TCR repertoires after primary sequences extraction from raw sequencing reads. This paper also provides comprehensive understanding of the diversity and dynamics indices for TCR sequencing data with serial time points and for comparing T cells in multiple compartments in a clinical context to ensure consistency and reproducibility of post-analysis. Tabular outputs and visualization tools with a simple enough R software usage enable scientists and clinicians with little computational experience to generate results in a well-presented format.

## Additional files

Additional file 1: Figure S6. Results of all ipilimumab treated prostate caner subjects and separately by long survivors (overall survival > = 23.6 months) and short survivors (overall survival < 23.6 months). (A) Shannon index of TCR at Week 0 and Week 2. (B) Clonality of TCR at Week 0 and Week 2. (C) intraclass correlation coefficient of TCR between Week 0 and Week 2. (D) Morisita’s distance of TCR between Week 0 and Week 2. (E) Scatter plot of Shannon vs. log10(# of uniques). Pearson correlation coefficient and corresponding pvalues were calculated. (F) Scatter plot of Clonality vs. log10(# of uniques). Pearson correlation coefficient and corresponding pvalues were calculated. (PDF 2080 kb)
Additional file 2: Figure S1. The diversity of TCR from PBMC at week 0, 2 and 4 for the healthy subjects (left) and the treated prostate cancer subjects (right) in NeoACT study. (A) The clonality of TCR from PBMC at week 0, 2 and 4 for the healthy subjects (left) and the treated prostate cancer subjects (right). (B) The geometric coefficient of variation (GCV) of TCR from PBMC at Week 0, 2 and 4 (PBMC.0, PBMC.2 and PBMC.4) for the healthy subjects (left) and the treated prostate cancer subjects (right). (PDF 1774 kb)
Additional file 3: Figure S2. The dynamics of TCR from PBMC across time course (NeoACT study). (A) The Baroni-Urbani and Buser (BUB) overlap index of TCR from PBMC across week 0, 2 and 4 (PBMC.0- > PBMC.2, PBMC.0- > PBMC.4 and PBMC.2- > PBMC.4) for the healthy subjects (left) and the treated prostate cancer subjects (right). (B) The intraclass correlation coefficient (ICC) of TCR from PBMC across week 0, 2 and 4 (PBMC.0- > PBMC.2, PBMC.0- > PBMC.4 and PBMC.2- > PBMC.4) for the healthy subjects (left) and the treated prostate cancer subjects (right). The ICC was calculated based on the clones present at both time points of each paired samples (i.e., the overlap clones). (C) A binned analysis of fold change in clonal frequency for the healthy subjects (left) and the treated prostate cancer subjects (right), for example, PBMC.0- > PBMC.2 is the fraction of clones where the ratio of frequencies at week 2 vs. week 0 is greater than 4 (“Increase”), less than 0.25 (“Decrease”), or between 0.25 and 4 (“Unchanged”), similarly for week 4 vs. week 0 (PBMC.0- > PBMC.4) and week 4 vs. week 2 (PBMC.2- > PBMC.4). This fold change analysis only includes the clones that present at both paired time points (i.e., the overlap clones). The median and interquartiles are shown. (PDF 1941 kb)
Additional file 4: Table S1. The results of serial 1 vs. 1 comparison by modified DESeq analysis for 5 treated prostate cancer subjects (24, 21, 16, 13, and 6). We considered 2 different ways of estimating dispersion: 1 vs. 1 uses Sample 1 and Sample 2 to calculate the dispersion; All Samples uses all available PBMC samples from 3 time points (PBMC.0, PBMC.2 and PBMC.4) to calculate the dispersion. Subject 6 doesn’t have data at week 4. The number of the significantly differentiated clones between Sample 1 and Sample 2 (FDR<0.05) was listed for each patient and each comparison. The summary statistics summarized across all 5 patients. **Table S2** The results of comparing PBMC samples of week 0 vs. week 2 and week 4, separately, by modified DESeq analysis for 5 treated prostate cancer subjects, where the dispersion was calculated based on all three PBMC samples. Overall mean stands for the average of log_10_(tissue count) of all the tissue-present clones. N is the number of the significantly differentiated (decreased or increased) clones (FDR<0.05). N* is the number of the significantly differentiated (decreased or increased) tissue-present clones. Mean stands for the average of log_10_(tissue count) of the corresponding N* significantly differentiated (decreased or increased) clones. P was obtained by comparing log_10_(tissue count) of the N* significantly differentiated (decreased or increased) tissue-present clones with the overall mean of log10(tissue count) using *t*-test. (DOCX 165 kb)
Additional file 5: Table S3. The list of bioinformatics tools to analyze high-throughput immunological repertoire sequencing data. (DOCX 174 kb)
Additional file 6: Robustness of Diversity/Dynamics Measures (Additional file 9). (DOCX 115 kb)
Additional file 7: Figure S4. The influence of the count thresholds on the pairwise binary similarity measures of TCR from PBMC at week 0, 2 and 4 for the treated prostate cancer subjects in NeoACT study. From top to bottom, each row shows five different types of similarity measures. From the left to the right, each column presents the different threshold of the clonotypes count (original data which is > =2, > = 5, > = 10, > = 15, > = 20, > = 25 and > =30). The subject with triangle shapes was the example used in Fig. 1c). The median and interquartiles are shown. (PDF 1234 kb)
Additional file 8: Figure S5. The influence of the count thresholds on the pairwise dynamics indices of TCR from PBMC at week 0, 2 and 4 for the treated prostate cancer subjects in NeoACT study. From top to bottom, each row shows the proportion of increase/unchanged/decrease clones from earlier time point to later time point, and pairwise Morisita’s distance. From the left to the right, each column presents the different threshold of the clonotypes count (original data which is > =2, > = 5, > = 10, > = 15, > = 20, > = 25 and > =30). The subject with triangle shapes was the example used in Fig. 1c). The median and interquartiles are shown. (PDF 1283 kb)
Additional file 9: Figure S3. The scatter plot of the number of unique clones with the Shannon index, Clonality and Geometric coefficient of variation (GCV) of TCR repertoire from PBMC (week 0, 2 and 4) of the five treated prostate cancer subjects in NeoACT study. Pearson correlation coefficient and corresponding pvalues were calculated for each pair. (PDF 2882 kb)

## Abbreviations

ANOVA: analysis of variance
BUB: Baroni-Urbani and Buser
CDR3: complementary determining region 3
CV: coefficient of variation
FC: fold change
FDR: false discovery rate
GCV: geometric coefficient of variation
ICC: intra-class correlation
NGS: next-generation sequencing
PBMC: peripheral blood mononuclear cell
TCR: T cell receptor
