# Supplementary material for: 3D: diversity, dynamics, differential testing – a proposed pipeline for analysis of next-generation sequencing T cell repertoire data
Source: BMC Bioinformatics. 2017 Feb 27;18:129. doi: 10.1186/s12859-017-1544-9 (PMC5327583; doi:10.1186/s12859-017-1544-9)

Supplementary Figure 6 **Results of Ipilimumab treated prostate caner subjects.**

(A) Shannon index of TCR at Week 0 and Week 2.

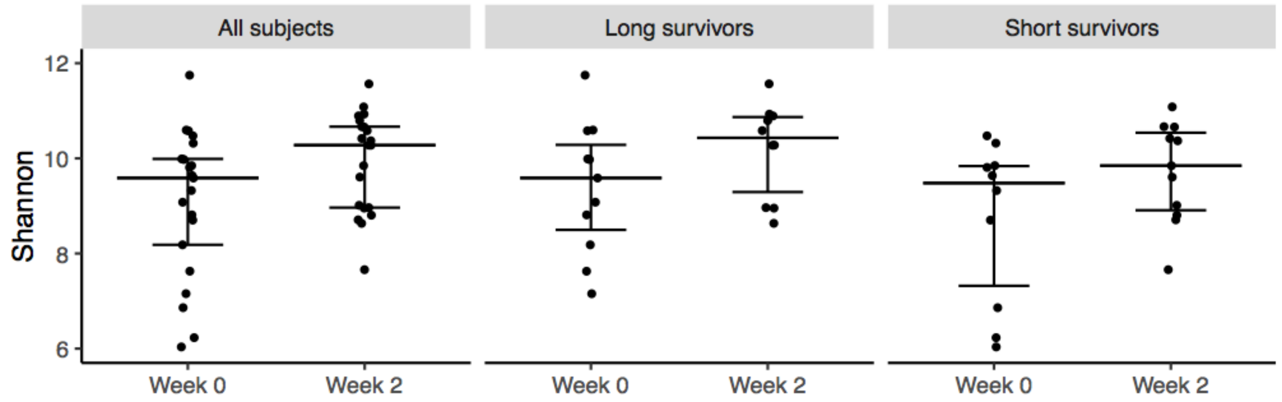

(B) Clonality of TCR at Week 0 and Week 2.

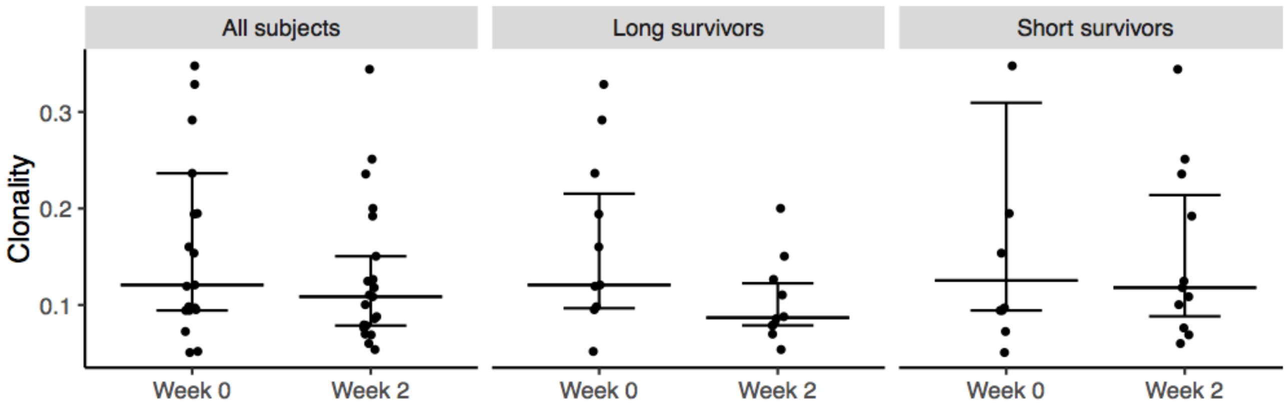

(C) Intraclass correlation coefficient of TCR between Week 0 and Week 2.

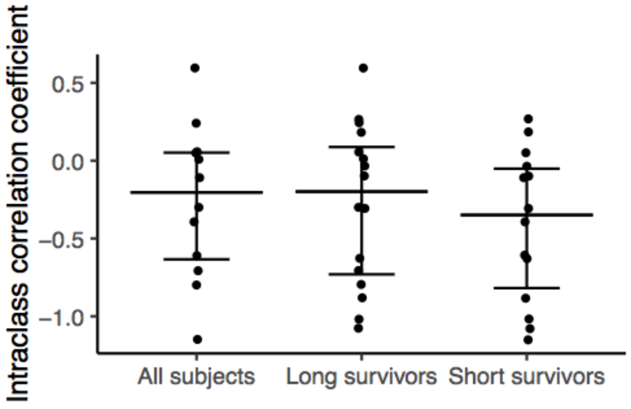

(D) Morisita distance of TCR between Week 0 and Week 2.

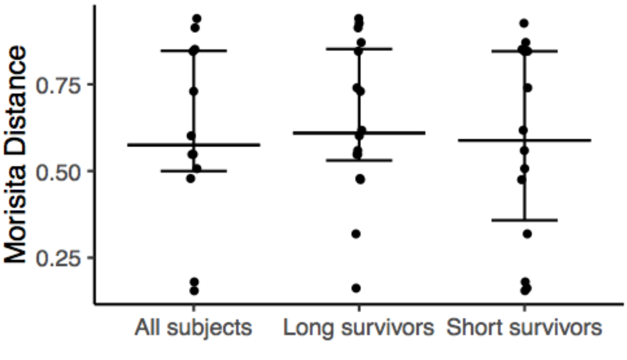

(E) Scatter plot of Shannon vs. $\log_{10}$(# of uniques)

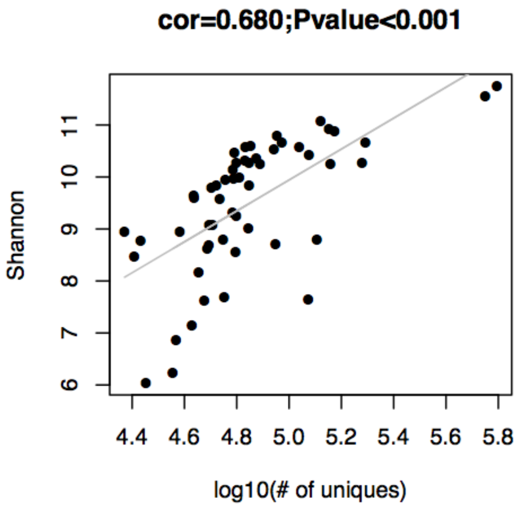

(F) Scatter plot of Clonality vs. $\log_{10}$(# of uniques).

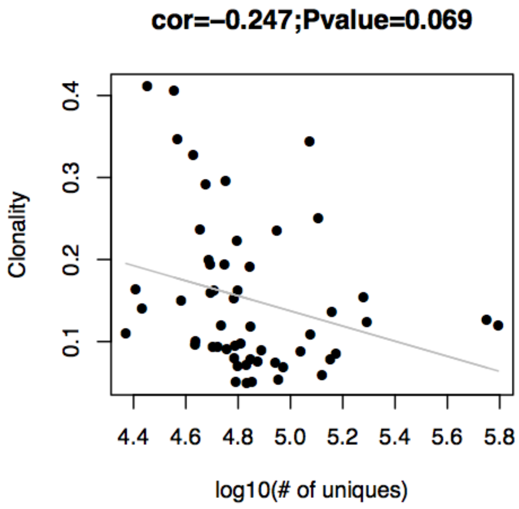

Supplement: Additional file 1: Figure S6. — Results of all ipilimumab treated prostate caner subjects and separately by long survivors (overall survival > = 23.6 months) and short survivors (overall survival < 23.6 months). (A) Shannon index of TCR at Week 0 and Week 2. (B) Clonality of TCR at Week 0 and Week 2. (C) intraclass correlation coefficient of TCR between Week 0 and Week 2. (D) Morisita’s distance of TCR between Week 0 and Week 2. (E) Scatter plot of Shannon vs. log10(# of uniques). Pearson correlation coefficient and corresponding pvalues were calculated. (F) Scatter plot of Clonality vs. log10(# of uniques). Pearson correlation coefficient and corresponding pvalues were calculated. (PDF 2080 kb) [file 12859_2017_1544_MOESM1_ESM.pdf]
