# Supplementary material for: 3D: diversity, dynamics, differential testing – a proposed pipeline for analysis of next-generation sequencing T cell repertoire data
Source: BMC Bioinformatics. 2017 Feb 27;18:129. doi: 10.1186/s12859-017-1544-9 (PMC5327583; doi:10.1186/s12859-017-1544-9)

Supplementary Figure 1 The diversity of TCR from PBMC at week 0, 2 and 4 for the healthy subjects (left) and the treated prostate cancer subjects (right) in NeoACT study .

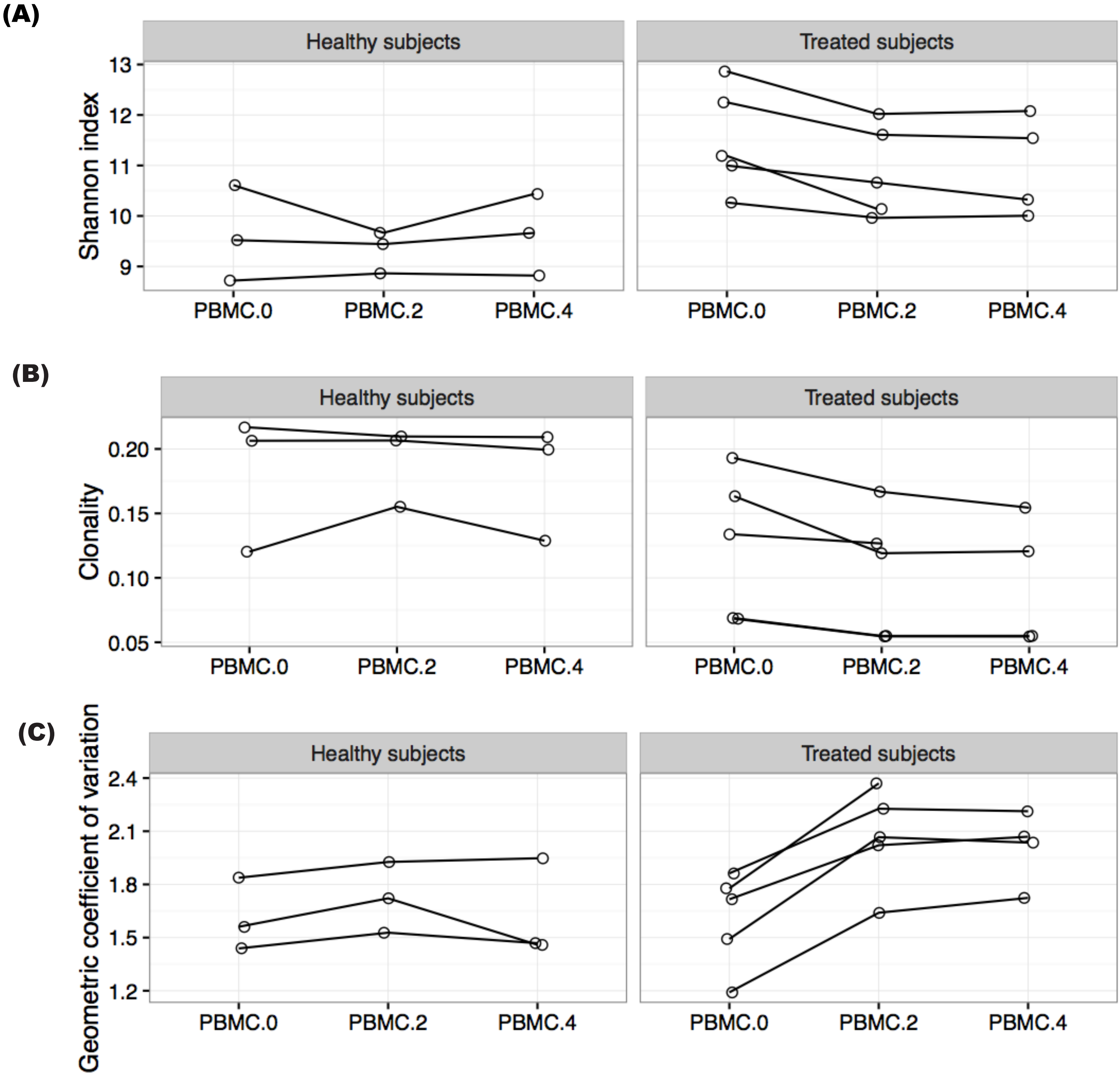

Supplement: Additional file 2: Figure S1. — The diversity of TCR from PBMC at week 0, 2 and 4 for the healthy subjects (left) and the treated prostate cancer subjects (right) in NeoACT study. (A) The clonality of TCR from PBMC at week 0, 2 and 4 for the healthy subjects (left) and the treated prostate cancer subjects (right). (B) The geometric coefficient of variation (GCV) of TCR from PBMC at Week 0, 2 and 4 (PBMC.0, PBMC.2 and PBMC.4) for the healthy subjects (left) and the treated prostate cancer subjects (right). (PDF 1774 kb) [file 12859_2017_1544_MOESM2_ESM.pdf]
