# Supplementary material for: 3D: diversity, dynamics, differential testing – a proposed pipeline for analysis of next-generation sequencing T cell repertoire data
Source: BMC Bioinformatics. 2017 Feb 27;18:129. doi: 10.1186/s12859-017-1544-9 (PMC5327583; doi:10.1186/s12859-017-1544-9)

Supplementary Figure 2 The dynamics in PBMC samples across time course (NeoACT study).

(A) The Baroni-Urbani and Buser (BUB) overlap index of TCR from PBMC across week 0, 2 and 4

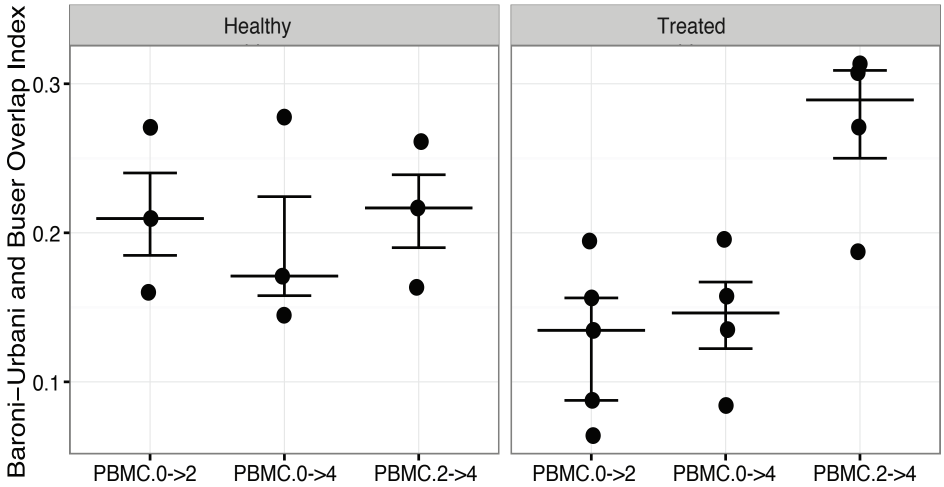

(B) The intraclass coefficient of TCR from PBMC across week 0, 2 and 4

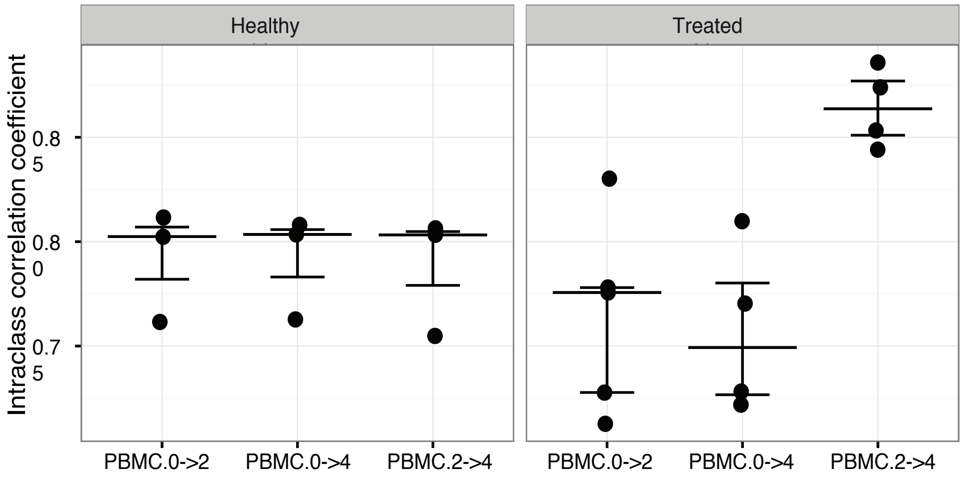

(C) A binned analysis of fold change in clonal frequency

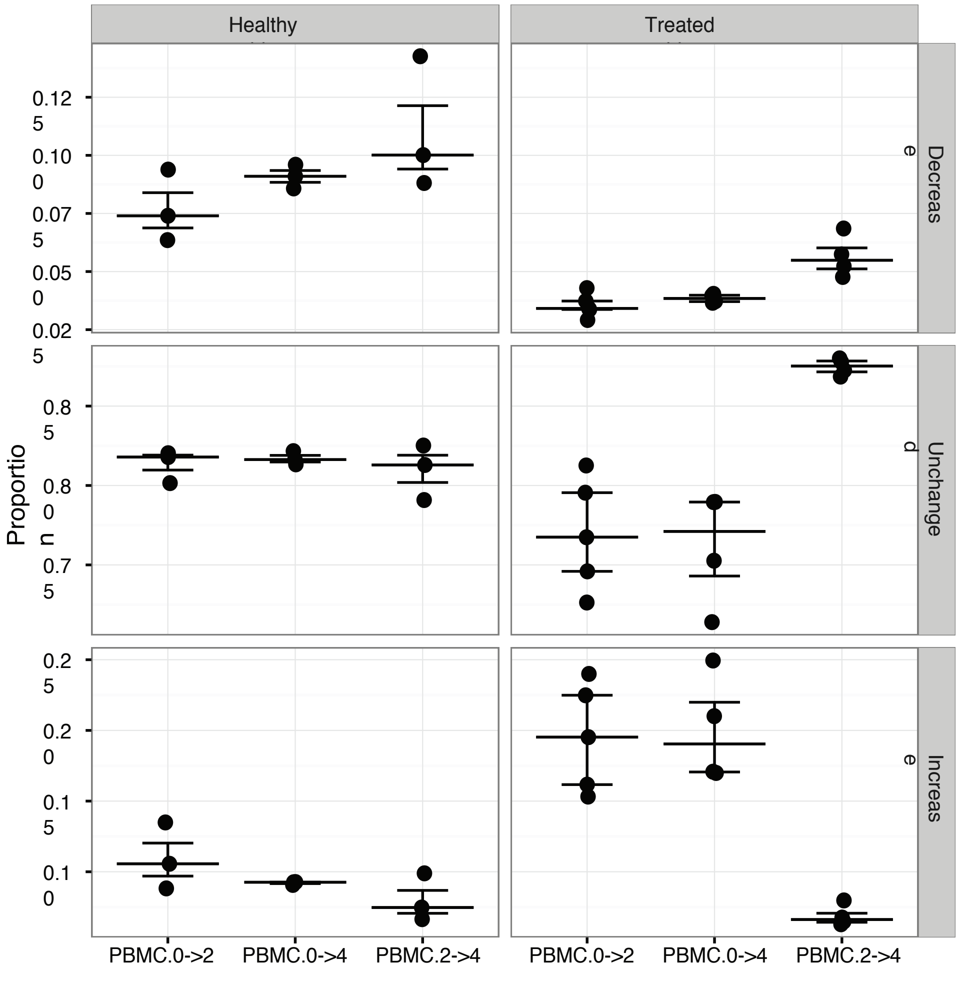

Supplement: Additional file 3: Figure S2. — The dynamics of TCR from PBMC across time course (NeoACT study). (A) The Baroni-Urbani and Buser (BUB) overlap index of TCR from PBMC across week 0, 2 and 4 (PBMC.0- > PBMC.2, PBMC.0- > PBMC.4 and PBMC.2- > PBMC.4) for the healthy subjects (left) and the treated prostate cancer subjects (right). (B) The intraclass correlation coefficient (ICC) of TCR from PBMC across week 0, 2 and 4 (PBMC.0- > PBMC.2, PBMC.0- > PBMC.4 and PBMC.2- > PBMC.4) for the healthy subjects (left) and the treated prostate cancer subjects (right). The ICC was calculated based on the clones present at both time points of each paired samples (i.e., the overlap clones). (C) A binned analysis of fold change in clonal frequency for the healthy subjects (left) and the treated prostate cancer subjects (right), for example, PBMC.0- > PBMC.2 is the fraction of clones where the ratio of frequencies at week 2 vs. week 0 is greater than 4 (“Increase”), less than 0.25 (“Decrease”), or between 0.25 and 4 (“Unchanged”), similarly for week 4 vs. week 0 (PBMC.0- > PBMC.4) and week 4 vs. week 2 (PBMC.2- > PBMC.4). This fold change analysis only includes the clones that present at both paired time points (i.e., the overlap clones). The median and interquartiles are shown. (PDF 1941 kb) [file 12859_2017_1544_MOESM3_ESM.pdf]
