# Supplementary material for: 3D: diversity, dynamics, differential testing – a proposed pipeline for analysis of next-generation sequencing T cell repertoire data
Source: BMC Bioinformatics. 2017 Feb 27;18:129. doi: 10.1186/s12859-017-1544-9 (PMC5327583; doi:10.1186/s12859-017-1544-9)
Supplement: Additional file 4: Table S1. — The results of serial 1 vs. 1 comparison by modified DESeq analysis for 5 treated prostate cancer subjects (24, 21, 16, 13, and 6). We considered 2 different ways of estimating dispersion: 1 vs. 1 uses Sample 1 and Sample 2 to calculate the dispersion; All Samples uses all available PBMC samples from 3 time points (PBMC.0, PBMC.2 and PBMC.4) to calculate the dispersion. Subject 6 doesn’t have data at week 4. The number of the significantly differentiated clones between Sample 1 and Sample 2 (FDR<0.05) was listed for each patient and each comparison. The summary statistics summarized across all 5 patients. Table S2 The results of comparing PBMC samples of week 0 vs. week 2 and week 4, separately, by modified DESeq analysis for 5 treated prostate cancer subjects, where the dispersion was calculated based on all three PBMC samples. Overall mean stands for the average of log10(tissue count) of all the tissue-present clones. N is the number of the significantly differentiated (decreased or increased) clones (FDR<0.05). N* is the number of the significantly differentiated (decreased or increased) tissue-present clones. Mean stands for the average of log10(tissue count) of the corresponding N* significantly differentiated (decreased or increased) clones. P was obtained by comparing log10(tissue count) of the N* significantly differentiated (decreased or increased) tissue-present clones with the overall mean of log10(tissue count) using t-test. (DOCX 165 kb) [file 12859_2017_1544_MOESM4_ESM.docx]

**Supplementary Table 1** The results of serial 1 vs. 1 comparison by modified DESeq analysis for 5 treated prostate cancer subjects (24, 21,16,13, and 6). We considered 2 different ways of estimating dispersion: **1 vs.1** uses Sample 1 and Sample 2 to calculate the dispersion; **All Samples** uses all available PBMC samples from 3 time points (PBMC.0, PBMC.2 and PBMC.4) to calculate the dispersion. Subject 6 doesn’t have data at week 4. The number of the significantly differentiated clones between Sample 1 and Sample 2 (FDR<0.05) was listed for each patient and each comparison. The summary statistics summarized across all 5 patients.

| **Dispersion Model** | **Sample 1** | **Sample 2** | **Subject ID** | | | | | **Summary Statistics** | | |
| --- | --- | --- | --- | --- | --- | --- | --- | --- | --- | --- |
|  |  |  | **24** | **21** | **16** | **13** | **6** | **Min** | **Median** | **Max** |
| **1 vs 1** | PBMC.0 | PBMC.2 | 60 | 6 | 56 | 50 | 41 | 6 | 50 | 60 |
|  | PBMC.0 | PBMC.4 | 69 | 15 | 119 | 10 | - | 10 | 42 | 119 |
|  | PBMC.2 | PBMC.4 | 37 | 18 | 72 | 16 | - | 16 | 27.5 | 72 |
| **All Samples** | PBMC.0 | PBMC.2 | 127 | 20 | 77 | 94 | - | 20 | 85.5 | 127 |
|  | PBMC.0 | PBMC.4 | 135 | 31 | 163 | 79 | - | 31 | 107 | 163 |
|  | PBMC.2 | PBMC.4 | 27 | 5 | 41 | 9 | - | 5 | 18 | 41 |

**Supplementary Table 2** The results of comparing PBMC samples of week 0 vs. week 2 and week 4, separately, by modified DESeq analysis for 5 treated prostate cancer subjects, where the dispersion was calculated based on all three PBMC samples. **Overall mean** stands for the average of log_10_(tissue count) of all the tissue-present clones. **N** is the number of the significantly differentiated (decreased or increased) clones (FDR<0.05). **N*** is the number of the significantly differentiated (decreased or increased) tissue-present clones. **Mean** stands for the average of log_10_(tissue count) of the corresponding N* significantly differentiated (decreased or increased) clones. **P** was obtained by comparing log_10_(tissue count) of the N* significantly differentiated (decreased or increased) tissue-present clones with the overall mean of log10(tissue count) using t-test.

| **Patient ID** | **Overall mean** | **Comparison** | **Significantly Differentiated** | | | | **Significantly Decreased** | | | | **Significantly Increased** | | | |
| --- | --- | --- | --- | --- | --- | --- | --- | --- | --- | --- | --- | --- | --- | --- |
|  |  |  | **N** | **N*** | **Mean** | **P** | **N** | **N*** | **Mean** | **P** | **N** | **N*** | **Mean** | **P** |
| 24 | 1.73 | PBMC.0 vs. PBMC.2 | 127 | 83 | 2.31 | <0.001 | 125 | 82 | 2.31 | <0.001 | 2 | 1 | 1.81 | NA |
|  |  | PBMC.0 vs. PBMC.4 | 135 | 89 | 2.31 | <0.001 | 134 | 89 | 2.31 | <0.001 | 1 | 0 | NA | NA |
| 21 | 1.78 | PBMC.0 vs. PBMC.2 | 20 | 13 | 2.59 | <0.001 | 19 | 12 | 2.55 | <0.001 | 1 | 1 | 3.04 | NA |
|  |  | PBMC.0 vs. PBMC.4 | 31 | 24 | 2.64 | <0.001 | 30 | 23 | 2.62 | <0.001 | 1 | 1 | 3.04 | NA |
| 16 | 1.93 | PBMC.0 vs. PBMC.2 | 77 | 52 | 2.54 | <0.001 | 77 | 52 | 2.54 | <0.001 | 0 | 0 | NA | NA |
|  |  | PBMC.0 vs. PBMC.4 | 163 | 112 | 2.51 | <0.001 | 161 | 112 | 2.51 | <0.001 | 2 | 0 | NA | NA |
| 13 | 1.83 | PBMC.0 vs. PBMC.2 | 94 | 33 | 2.16 | 0.002 | 93 | 32 | 2.16 | 0.003 | 1 | 1 | 2.16 | NA |
|  |  | PBMC.0 vs. PBMC.4 | 79 | 34 | 2.29 | <0.001 | 76 | 33 | 2.28 | <0.001 | 3 | 1 | 2.80 | NA |
| 6 | 1.78 | PBMC.0 vs. PBMC.2 | 41 | 17 | 2.08 | 0.091 | 40 | 17 | 2.08 | 0.091 | 1 | 0 | NA | NA |
|  |  | PBMC.0 vs. PBMC.4 | NA | NA | NA | NA | NA | NA | NA | NA | NA | NA | NA | NA |
