# Supplementary material for: 3D: diversity, dynamics, differential testing – a proposed pipeline for analysis of next-generation sequencing T cell repertoire data
Source: BMC Bioinformatics. 2017 Feb 27;18:129. doi: 10.1186/s12859-017-1544-9 (PMC5327583; doi:10.1186/s12859-017-1544-9)
Supplement: Additional file 5: Table S3. — The list of bioinformatics tools to analyze high-throughput immunological repertoire sequencing data. (DOCX 174 kb) [file 12859_2017_1544_MOESM5_ESM.docx]

**Supplementary Table 3** The list of bioinformatics tools to analyze high-throughput immunological repertoire sequencing data [1].

| **Tools** | **Preprocess^[[1]](#footnote-1)^** | **Downstream Analysis** | | |
| --- | --- | --- | --- | --- |
|  |  | Diversity | Dynamics | Differential Testing |
| 3D Analysis |  | Yes | Yes | Yes |
| AbMining ToolBox [2] | Yes |  |  |  |
| ARResT/Interrogate[3] | Yes |  |  |  |
| BASELINe [4, 5] | Yes |  |  |  |
| Change-O [6] | Yes | Yes |  |  |
| clonotypeR [7] | Yes |  |  |  |
| Decombinator [8] | Yes |  |  |  |
| HTJoinSolver | Yes |  |  |  |
| IgBLAST [9] | Yes |  |  |  |
| IGGalaxy [10] | Yes |  |  |  |
| IgRepertoireConstructor [11] | Yes |  |  |  |
| IMEX/IMmunEXplorer[12] | Yes | Yes |  |  |
| IMGT/V-QUEST [13-15] | Yes |  |  |  |
| IMonitor [16] | Yes | Yes |  |  |
| ImmuneDB[17] | Yes |  |  |  |
| ImmunediveRsity [18] | Yes | Yes |  |  |
| ImmunoSEQ Analyzer [19] | Yes | Yes | Yes |  |
| IMSEQ [20] | Yes |  |  |  |
| LymAnalyzer [21] | Yes |  |  |  |
| MiGEC [22] | Yes |  |  |  |
| MiTCR [23] | Yes |  |  |  |
| MiXCR [24] | Yes |  |  |  |
| Partis [25] | Yes |  |  |  |
| pRESTO [26] | Yes |  |  |  |
| repgenHMM [27] | Yes | Yes |  |  |
| RTCR/Recover TCR [28] | Yes |  |  |  |
| sciReptor [29] | Yes |  |  |  |
| tcR [30] | Yes | Yes | Yes |  |
| TCRklass [31] | Yes |  |  |  |
| TIgGER [32] | Yes |  |  |  |
| TraCeR [33] | Yes |  |  |  |
| TRIgS [34] | Yes |  |  |  |
| VDJ [35] | Yes |  |  |  |
| VDJFasta [36] | Yes |  |  |  |
| VDJPuzzle [37] | Yes |  |  |  |
| VDJSeq-Solver [38] | Yes |  |  |  |
| VDJtools[39] | Yes | Yes |  |  |
| Vigil [40] | Yes |  |  |  |

**Reference:**

1. <https://omictools.com/rep-seq-category>, Accessed 15 December 2016.

2. D'Angelo, S., et al., *The antibody mining toolbox: an open source tool for the rapid analysis of antibody repertoires.* MAbs, 2014. **6**(1): p. 160-72.

3. Bystry, V., et al., *ARResT/Interrogate: an interactive immunoprofiler for IG/TR NGS data.* Bioinformatics, 2016.

4. Uduman, M., et al., *Detecting selection in immunoglobulin sequences.* Nucleic Acids Res, 2011. **39**(Web Server issue): p. W499-504.

5. Yaari, G., M. Uduman, and S.H. Kleinstein, *Quantifying selection in high-throughput Immunoglobulin sequencing data sets.* Nucleic Acids Res, 2012. **40**(17): p. e134.

6. Gupta, N.T., et al., *Change-O: a toolkit for analyzing large-scale B cell immunoglobulin repertoire sequencing data.* Bioinformatics, 2015. **31**(20): p. 3356-8.

7. Plessy, C.M.-F., E; Manabe, RI; Hori, S., *clonotypeR--high throughput analysis of T cell antigen receptor sequences.* bioRxiv. **doi:** <https://doi.org/10.1101/028696>.

8. Thomas, N., et al., *Decombinator: a tool for fast, efficient gene assignment in T-cell receptor sequences using a finite state machine.* Bioinformatics, 2013. **29**(5): p. 542-550.

9. Ye, J., et al., *IgBLAST: an immunoglobulin variable domain sequence analysis tool.* Nucleic Acids Res, 2013. **41**(Web Server issue): p. W34-40.

10. Moorhouse, M.J., et al., *ImmunoGlobulin galaxy (IGGalaxy) for simple determination and quantitation of immunoglobulin heavy chain rearrangements from NGS.* BMC Immunol, 2014. **15**: p. 59.

11. Safonova, Y., et al., *IgRepertoireConstructor: a novel algorithm for antibody repertoire construction and immunoproteogenomics analysis.* Bioinformatics, 2015. **31**(12): p. i53-61.

12. Schaller, S., et al., *ImmunExplorer (IMEX): a software framework for diversity and clonality analyses of immunoglobulins and T cell receptors on the basis of IMGT/HighV-QUEST preprocessed NGS data.* BMC Bioinformatics, 2015. **16**: p. 252.

13. Giudicelli, V., D. Chaume, and M.P. Lefranc, *IMGT/V-QUEST, an integrated software program for immunoglobulin and T cell receptor V-J and V-D-J rearrangement analysis.* Nucleic Acids Res, 2004. **32**(Web Server issue): p. W435-40.

14. Giudicelli, V., X. Brochet, and M.P. Lefranc, *IMGT/V-QUEST: IMGT standardized analysis of the immunoglobulin (IG) and T cell receptor (TR) nucleotide sequences.* Cold Spring Harb Protoc, 2011. **2011**(6): p. 695-715.

15. Brochet, X., M.P. Lefranc, and V. Giudicelli, *IMGT/V-QUEST: the highly customized and integrated system for IG and TR standardized V-J and V-D-J sequence analysis.* Nucleic Acids Res, 2008. **36**(Web Server issue): p. W503-8.

16. Zhang, W., et al., *IMonitor: A Robust Pipeline for TCR and BCR Repertoire Analysis.* Genetics, 2015. **201**(2): p. 459-+.

17. Rosenfeld, A.M., et al., *ImmuneDB: A system for the analysis and exploration of high-throughput adaptive immune receptor sequencing data.* Bioinformatics, 2016.

18. Cortina-Ceballos, B., et al., *Reconstructing and mining the B cell repertoire with ImmunediveRsity.* MAbs, 2015. **7**(3): p. 516-24.

19. <http://www.adaptivebiotech.com/immunoseq/analyzer>, Accessed 1 December 2016.

20. Kuchenbecker, L., et al., *IMSEQ--a fast and error aware approach to immunogenetic sequence analysis.* Bioinformatics, 2015. **31**(18): p. 2963-71.

21. Yu, Y., R. Ceredig, and C. Seoighe, *LymAnalyzer: a tool for comprehensive analysis of next generation sequencing data of T cell receptors and immunoglobulins.* Nucleic Acids Res, 2016. **44**(4): p. e31.

22. Shugay, M., et al., *Towards error-free profiling of immune repertoires.* Nat Methods, 2014. **11**(6): p. 653-5.

23. Bolotin, D.A., et al., *MiTCR: software for T-cell receptor sequencing data analysis.* Nature Methods, 2013. **10**(9): p. 813-814.

24. Bolotin, D.A., et al., *MiXCR: software for comprehensive adaptive immunity profiling.* Nature Methods, 2015. **12**(5): p. 380-381.

25. Ralph, D.K. and F.A.t. Matsen, *Consistency of VDJ Rearrangement and Substitution Parameters Enables Accurate B Cell Receptor Sequence Annotation.* PLoS Comput Biol, 2016. **12**(1): p. e1004409.

26. Vander Heiden, J.A., et al., *pRESTO: a toolkit for processing high-throughput sequencing raw reads of lymphocyte receptor repertoires.* Bioinformatics, 2014. **30**(13): p. 1930-2.

27. Elhanati, Y., et al., *repgenHMM: a dynamic programming tool to infer the rules of immune receptor generation from sequence data.* Bioinformatics, 2016. **32**(13): p. 1943-51.

28. Gerritsen, B., et al., *RTCR: a pipeline for complete and accurate recovery of T cell repertoires from high throughput sequencing data.* Bioinformatics, 2016. **32**(20): p. 3098-3106.

29. Imkeller, K., et al., *sciReptor: analysis of single-cell level immunoglobulin repertoires.* BMC Bioinformatics, 2016. **17**: p. 67.

30. Nazarov, V.I., et al., *tcR: an R package for T cell receptor repertoire advanced data analysis.* BMC Bioinformatics, 2015. **16**: p. 175.

31. Yang, X., et al., *TCRklass: a new K-string-based algorithm for human and mouse TCR repertoire characterization.* J Immunol, 2015. **194**(1): p. 446-54.

32. Gadala-Maria, D., et al., *Automated analysis of high-throughput B-cell sequencing data reveals a high frequency of novel immunoglobulin V gene segment alleles.* Proc Natl Acad Sci U S A, 2015. **112**(8): p. E862-70.

33. Stegle, O., S.A. Teichmann, and J.C. Marioni, *Computational and analytical challenges in single-cell transcriptomics.* Nat Rev Genet, 2015. **16**(3): p. 133-45.

34. Lees, W.D. and A.J. Shepherd, *Utilities for High-Throughput Analysis of B-Cell Clonal Lineages.* J Immunol Res, 2015. **2015**: p. 323506.

35. Laserson, U., et al., *High-resolution antibody dynamics of vaccine-induced immune responses.* Proc Natl Acad Sci U S A, 2014. **111**(13): p. 4928-33.

36. Glanville, J., et al., *Precise determination of the diversity of a combinatorial antibody library gives insight into the human immunoglobulin repertoire.* Proceedings of the National Academy of Sciences of the United States of America, 2009. **106**(48): p. 20216-20221.

37. Eltahla, A.A., et al., *Linking the T cell receptor to the single cell transcriptome in antigen-specific human T cells.* Immunol Cell Biol, 2016. **94**(6): p. 604-11.

38. Paciello, G., et al., *VDJSeq-Solver: in silico V(D)J recombination detection tool.* PLoS One, 2015. **10**(3): p. e0118192.

39. Shugay, M., et al., *VDJtools: Unifying Post-analysis of T Cell Receptor Repertoires.* PLoS Comput Biol, 2015. **11**(11): p. e1004503.

40. Giraud, M., et al., *Fast multiclonal clusterization of V(D)J recombinations from high-throughput sequencing.* BMC Genomics, 2014. **15**: p. 409.

1. Preprocess includes but not limited to mapping antigen receptor segments to sequencing reads and assembling the clonotypes and extracting CDRs. [↑](#footnote-ref-1)
