# Supplementary material for: 3D: diversity, dynamics, differential testing – a proposed pipeline for analysis of next-generation sequencing T cell repertoire data
Source: BMC Bioinformatics. 2017 Feb 27;18:129. doi: 10.1186/s12859-017-1544-9 (PMC5327583; doi:10.1186/s12859-017-1544-9)
Supplement: Additional file 6: — Robustness of Diversity/Dynamics Measures (Additional file 9). (DOCX 115 kb) [file 12859_2017_1544_MOESM6_ESM.docx]

**Robustness of Diversity/Dynamics Measures**

To explore the robustness of the diversity index, we calculated the correlation coefficient of each diversity index with log_10_(number of unique clones) by using TCR sequencing data from PBMC at all time points of the 5 treated subjects of NeoACT study (Supplementary Fig 3). We found that both the Shannon index and GCV were significantly correlated with the number of unique clones (p=0.001 and p<0.001, respectively) with large correlations (r=0.77 and -0.95, respectively), however, the clonality was not significantly correlated to the number of unique clones (p=0.38, r=-0.25). Similar results were observed for the prostate cancer patients treated by ipilimumab combined GM-CSF (Supplementary Fig 6).

The original TCR data was filtered by count$\geq$2, i.e., any clones that have count <2 were excluded. As mentioned earlier a frequency count of 10 to 30 might be a pivot point (Fig 1C), and clones with a very low frequency count (e.g., <5) might not be biologically important. We thus applied different filtering thresholds ($\geq$5, $\geq$10, $\geq$15, $\geq$20, $\geq$25 and $\geq$30) when exploring stability of the diversity index by normalizing the frequency of each clone. As shown in Fig 5A, Clonality was the only diversity index that displayed a stable profile across the time points for each of the thresholds, while the patterns of the Shannon index changed from a decreasing to increasing profile as the threshold was increased. Since the values of Gini Simpson index shrink to 0.995-1 for smaller threshold and the range of inverse Simpson is extremely wide, it’s difficult to draw s stable and meaningful inference based on either index. In comparison, the decreasing GCV pattern became invisible with an increased threshold value. In addition, pairwise relative clonality (Fig 5B) were calculated as Clonality of PBMC at the later time point divided by that of the earlier time point, e.g., PBMC.2/0 = Clonality of week 2 PBMC divided by week 0 PBMC. Across different thresholds, the relative clonality of week 4 vs. week 2 (PBMC.4/2) was constantly close to 1, while the relative clonality of week 2 vs. week 0 (PBMC.2/0) and week 4 vs. week 0 (PBMC.4/0) was always smaller than 1. Therefore, Clonality was suggested to be the first choice to measure the TCR repertoire diversity.

Supplementary Fig 4 shows the influence of thresholds on the binary similarity measures. Overall all the binary similarity measures have less distinguish with the increase of the threshold and the direction of the difference starts to change after threshold of 20. This implies that the relative low overlap between week 2 (4) and week 0 is caused by the big proportion of low count clones (<20) of the TCR repertoire at week 0. Therefore, the Sip-T treatment effect in TCR repertoire might mainly present in those low-count clones and simply focusing the analysis on the top rank clones might not be appropriate. Similarly, the similar phenomena were observed when characterizing the clones into increase/unchanged/decrease groups (Supplementary Fig 4). However, Morisita’s distance seems more distinguished with the increased threshold. Though Morisita’s distance is based on the assumption that increasing the size of the samples will increase the diversity because it would include more different clonotypes, if the low-count clones occupy a big proportion of the TCR repertoire, then they might shade the light of the major real diversity of the TCR repertoire.
