# Supplementary material for: 3D: diversity, dynamics, differential testing – a proposed pipeline for analysis of next-generation sequencing T cell repertoire data
Source: BMC Bioinformatics. 2017 Feb 27;18:129. doi: 10.1186/s12859-017-1544-9 (PMC5327583; doi:10.1186/s12859-017-1544-9)

Supplementary Figure 5 The influence of the count thresholds on the pairwise dynamic indices of TCR from PBMC at week 0, 2 and 4 for the treated prostate cancer subjects subjects in NeoACT study.

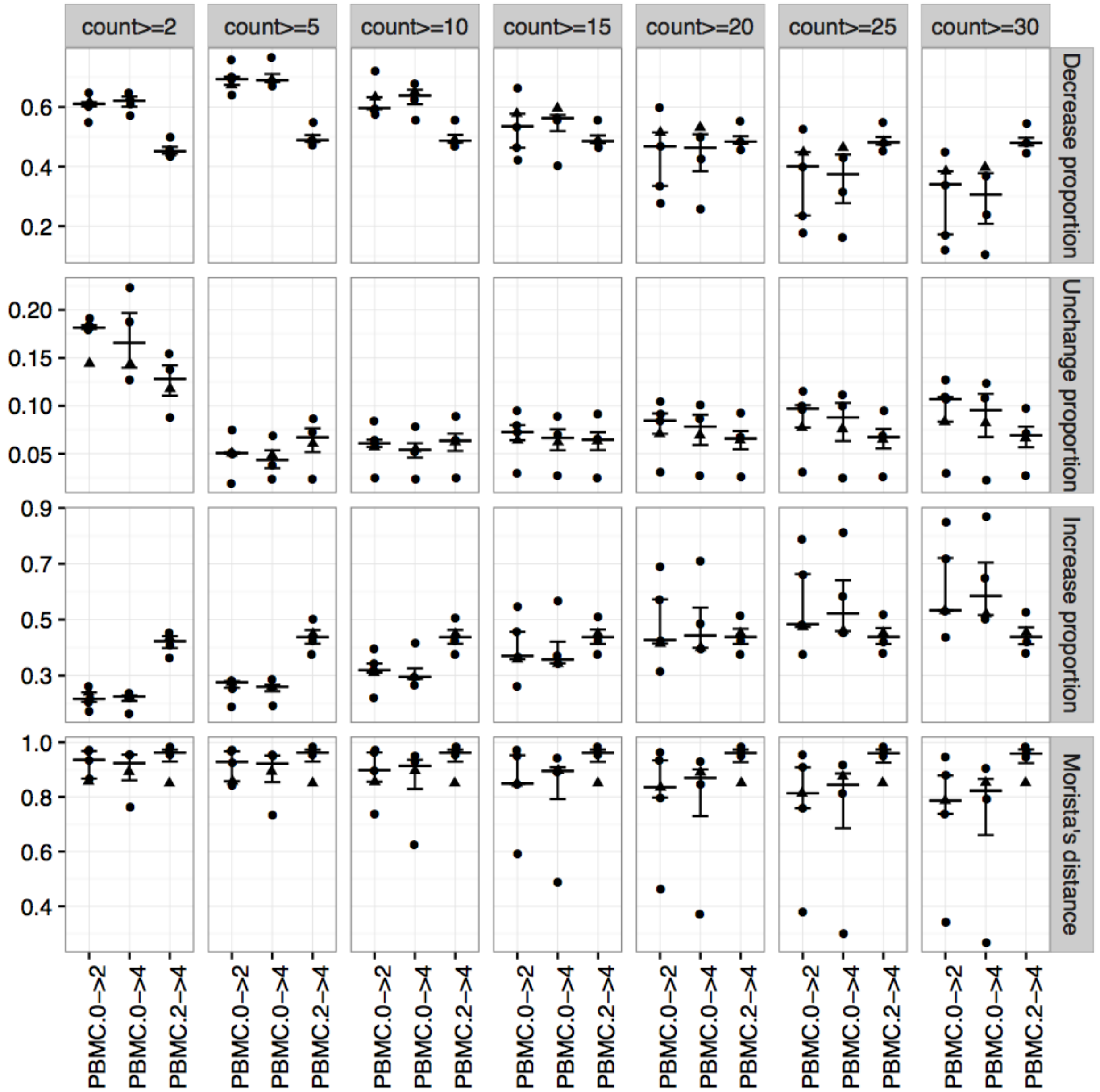

Supplement: Additional file 8: Figure S5. — The influence of the count thresholds on the pairwise dynamics indices of TCR from PBMC at week 0, 2 and 4 for the treated prostate cancer subjects in NeoACT study. From top to bottom, each row shows the proportion of increase/unchanged/decrease clones from earlier time point to later time point, and pairwise Morisita’s distance. From the left to the right, each column presents the different threshold of the clonotypes count (original data which is > =2, > = 5, > = 10, > = 15, > = 20, > = 25 and > =30). The subject with triangle shapes was the example used in Fig. 1c). The median and interquartiles are shown. (PDF 1283 kb) [file 12859_2017_1544_MOESM8_ESM.pdf]
