# Supplementary material for: 3D: diversity, dynamics, differential testing – a proposed pipeline for analysis of next-generation sequencing T cell repertoire data
Source: BMC Bioinformatics. 2017 Feb 27;18:129. doi: 10.1186/s12859-017-1544-9 (PMC5327583; doi:10.1186/s12859-017-1544-9)

Supplementary Figure 3 **The scatter plot of the number of unique clones with the Shannon index, Clonality and Geometric coefficient of variation (GCV) of TCR repertoire from PBMC and FP (week 0, 2 and 4) of the five treated prostate cancer subjects in NeoACT study.**

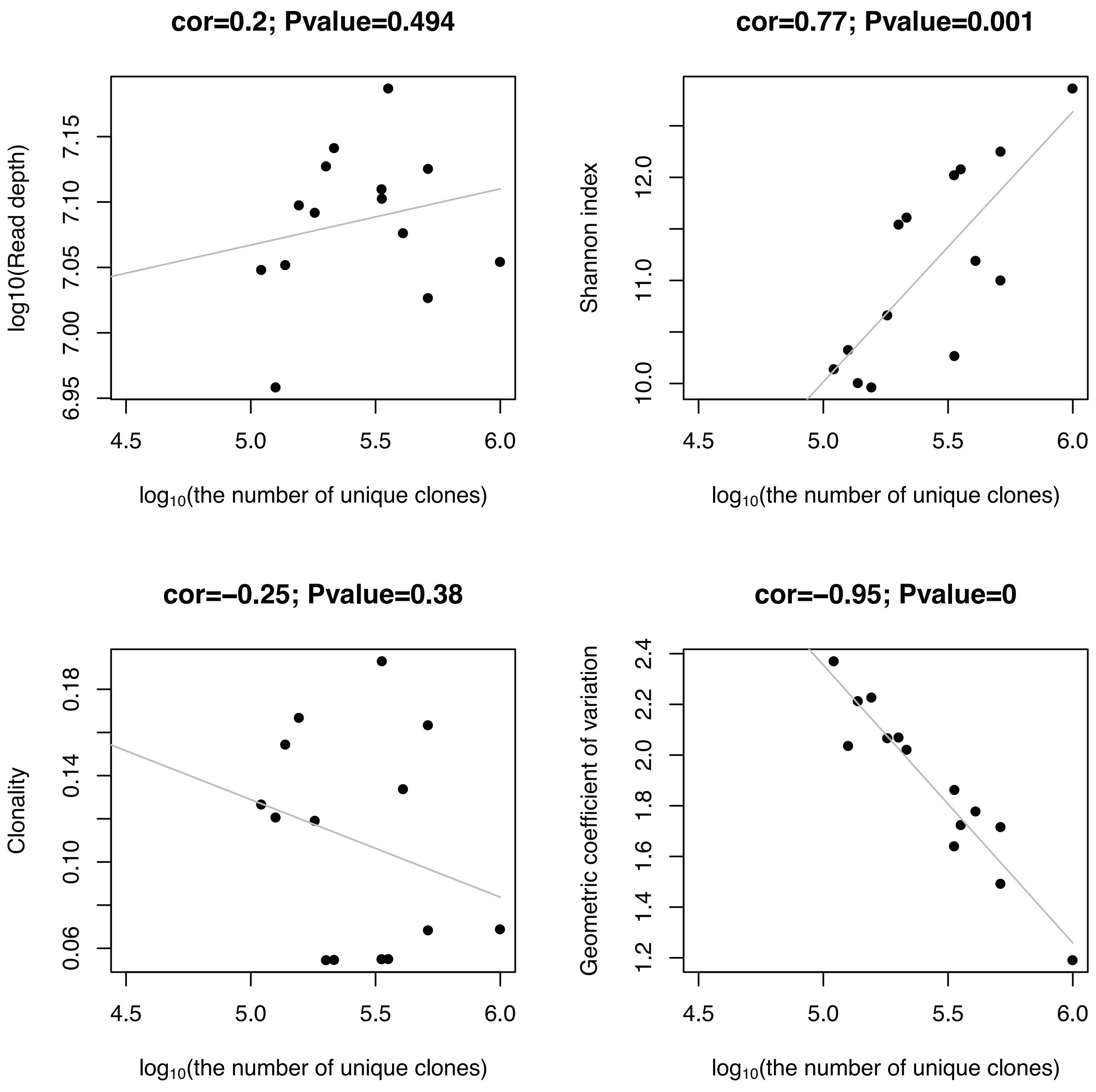

Supplement: Additional file 9: Figure S3. — The scatter plot of the number of unique clones with the Shannon index, Clonality and Geometric coefficient of variation (GCV) of TCR repertoire from PBMC (week 0, 2 and 4) of the five treated prostate cancer subjects in NeoACT study. Pearson correlation coefficient and corresponding pvalues were calculated for each pair. (PDF 2882 kb) [file 12859_2017_1544_MOESM9_ESM.pdf]
